# Single-cell transcriptome analysis demonstrates inter-patient and intra-tumor heterogeneity in primary and metastatic lung adenocarcinoma

**DOI:** 10.18632/aging.103945

**Published:** 2020-11-10

**Authors:** Yafei Liu, Guanchao Ye, Lan Huang, Chunyang Zhang, Yinliang Sheng, Bin Wu, Lu Han, Chunli Wu, Bo Dong, Yu Qi

**Affiliations:** 1 Department of Thoracic Surgery, The First Affiliated Hospital of Zhengzhou University, Zhengzhou 450052, China; 2 Biotherapy Center, The First Affiliated Hospital of Zhengzhou University, Zhengzhou 450052, China

**Keywords:** lung adenocarcinoma, single cell RNA sequencing, tumour heterogeneity, chemoresistance

## Abstract

In this study, we performed single-cell transcriptome data analysis of fifty primary and metastatic lung adenocarcinoma (LUAD) samples from the GSE123902 and GSE131907 datasets to determine the landscape of inter-patient and intra-tumoral heterogeneity. The gene expression profiles and copy number variations (CNV) showed significant heterogeneity in the primary and metastatic LUAD samples. We observed upregulation of pathways related to translational initiation, endoplasmic reticulum stress, exosomes, and unfolded protein response in the brain metastasis samples as compared to the primary tumor samples. Pathways related to exosomes, cell adhesion and metabolism were upregulated and the epithelial-to-mesenchymal-transition (EMT) pathway was downregulated in brain metastasis samples from chemotherapy-treated LUAD patients as compared to those from the untreated LUAD patients. Tumor cell subgroups in the brain metastasis samples showed differential expression of genes related to type II alveolar cells, chemoresistance, glycolysis and oxidative phosphorylation (metabolic reprogramming), and EMT. Thus, single-cell transcriptome analysis demonstrated intra-patient and intra-tumor heterogeneity in the regulation of pathways related to tumor progression, chemoresistance and metabolism in the primary and metastatic LUAD tissues. Moreover, our study demonstrates that single cell transcriptome analysis is a potentially useful tool for accurate diagnosis and personalized targeted treatment of LUAD patients.

## INTRODUCTION

Lung cancer is the leading cause of cancer incidence and mortality, accounting for nearly 2.1 million new lung cancer cases and 1.8 million deaths worldwide in 2018 [1]. The two main subtypes of lung cancer are small-cell lung carcinoma and non-small-cell lung carcinoma (NSCLC), which account for 15% and 85% of all lung cancer cases, respectively [2]. NSCLC is further classified into squamous cell carcinoma, adenocarcinoma, and large cell carcinoma. Adenocarcinoma is the most common type of lung cancer that accounts for approximately 40% of all lung cancer cases [3]. The prognosis of lung cancer patients and their treatment is mainly based on the pathological stage of the disease [4]. There have been great advances in the clinical diagnosis and treatment of NSCLC, but the prognosis remains poor [5]. Currently, the standard therapy for clinical stage IA non–small cell lung cancer is surgical resection with lobectomy and mediastinal lymph node (LN) staging [6]. The treatment for advanced lung adenocarcinoma patients includes targeted therapy and chemo-radiotherapy [7]. Treatment with epidermal growth factor receptor (EGFR)-tyrosine kinase inhibitors is more effective for patients with EGFR-mutated lung adenocarcinoma and acceptable for EGFR-mutated NSCLC with brain metastases [8]. However, there is great scope for developing novel therapies to improve survival outcomes in NSCLC patients, especially those with site-specific metastases [9]. Combinatorial therapies show better clinical outcomes because they simultaneously block multiple cancer-related signaling pathways, including those related to drug resistance [10]. Intratumor heterogeneity has major implications for diagnosis and therapy of solid cancers because a single tumor biopsy may not provide complete information regarding the molecular characteristics of the primary and metastatic tumors [11]. Hence, dissecting the clonal composition of tumors at a genetic level is essential for the understanding of the biological nature and the developmental status of the cancer and subsequently assess prognosis and design effective treatment strategy [12]. Single-cell genome profiling technology provides the highest sensitivity in analyzing the intra-tumoral genetic heterogeneity and provides an understanding of the biological processes that are activated or suppressed in different clonal populations of tumor cells, which can then guide personalized targeted treatment strategy for individual cancer patients [13]. In this study, we performed single-cell RNA sequencing data analysis of the GEO LUAD patient datasets to determine intra-patient and intra-tumoral heterogeneity. We also aimed to identify specific molecular signatures related to tumor progression and chemoresistance that can be relevant in identifying potential therapeutic targets and designing personalized therapeutic regimens to improve survival outcomes of LUAD patients.

## RESULTS

### Analysis of single-cell transcriptomic profiles from primary LUAD and brain metastasis tissue samples

**Figure 1:**
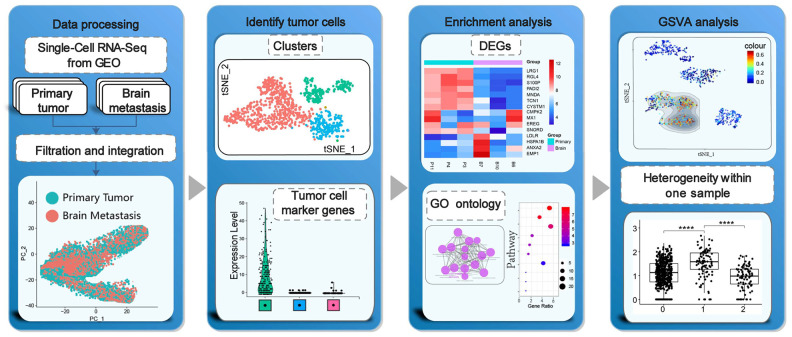
**Schematic representation of the study strategy.** Our study strategy included quality control, data integration and filtration of transcriptome data, cell clustering and identification of heterogeneous tumor cells from the same tumor samples, functional enrichment analysis, gene set variation analysis (GSVA), and tumor cell subgroup analysis.

**Figure 2:**
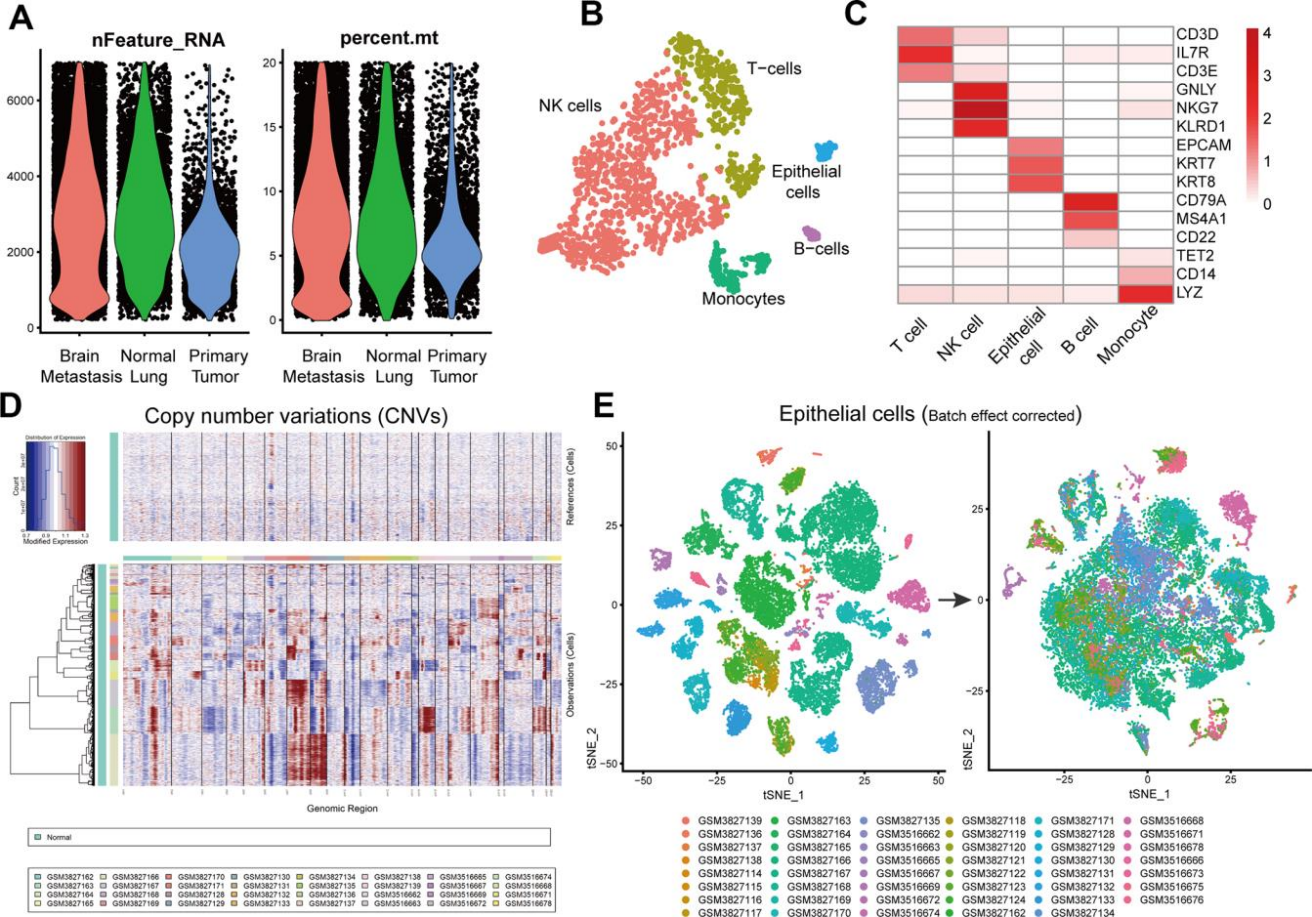
**Identification of normal lung epithelial cells and tumor cells.** (**A**) Violin plots show genes numbers and the percentage of mitochondrial genome per single cell from the primary LUAD and brain metastatic tissues, and the normal lung tissues from the GSE123902 [13] and GSE131907 [14] datasets. (**B**) The tSNE plot demonstrates five different cell types in a single non-tumor lung tissue sample and highlights annotation accuracy of the Single R package analysis. (**C**) Heatmap shows the expression of marker genes in different cell types from a single non-tumor lung tissue sample. (**D**) InferCNV plot shows diverse chromosomal copy number variation (CNVs) in the tumor cells from primary and metastatic LUAD tissue samples. Normal lung tissue samples are used as controls. (**E**) Seurat analysis results with batch effect correction after integrating primary and metastatic LUAD and normal lung epithelial cells.

The overall study strategy is shown in Figure 1. We analyzed 14 primary LUAD, metastatic LUAD and normal lung tissue samples from the GSE123902 dataset [14] and 36 primary LUAD, metastatic LUAD and normal lung tissue samples from the GSE131907 dataset [15]. Overall, we obtained 170831 single-cell transcriptomic profiles, including 80002 from primary LUAD tissue samples, 35364 from brain metastasis tissue samples, and 55465 from normal lung tissue samples We analyzed the sample characteristics and retained cells that showed >200 and <7000 genes as well <20% mitochondrial genome reads (Figure 2A). We integrated the transcriptome data from 108892 tumor cells belonging to primary LUAD or brain metastatic tissues and 55241 cells from non-tumor lung samples into the Seurat object (https://satijalab.org/seurat/) The single R algorithm annotated 4014 cells from non-tumor lung tissues and 22200 cells from primary LUAD and brain metastasis tissues as epithelial cells. We confirmed the accuracy of the SingleR cell annotation by comparing the expression of several known marker genes in the non-tumor lung tissues (Figure 2B, 2C). Furthermore, we integrated the data from all cells and analyzed the copy number variations (CNV) using the inferCNV R package (Figure 2D). Based on these analyses, we designated the cell clusters with mostly normal lung cells as normal lung epithelial cells, whereas the remaining cell clusters with LUAD-related lesions were designated as tumor cells for joint analysis. Moreover, we tested several epithelial tumor marker genes using Seurat and Single Cell Signature Explorer packages (Supplementary Figure 1). Then, we integrated the tumor and normal lung epithelial cells and performed batch correction analysis using Seurat (Figure 2E). The general comparison methods between samples are shown in Supplementary Table 1.

We analyzed the single-cell RNA-seq data using inferCNV R package to detect amplifications or deletions in chromosomes and identified several copy number variations (CNVs) in different chromosomes in the tumor samples (Figure 2D). For example, the primary LUAD sample, GSM3516669, showed amplifications and deletions in chromosome 1 and deletions in chromosome 19. Another primary LUAD sample, GSM3516662, showed small amplifications in chromosome 1. The brain metastasis sample GSM3516671, showed amplifications in chromosomes 1, 2, 12, and 20, and deletions on chromosomes 6, 8, 13, and 19. These CNV results demonstrated clear-cut differences between tumor cells and normal epithelial cells (controls).

### Landscape of gene expression heterogeneity in primary and brain metastasis LUAD samples

**Figure 3:**
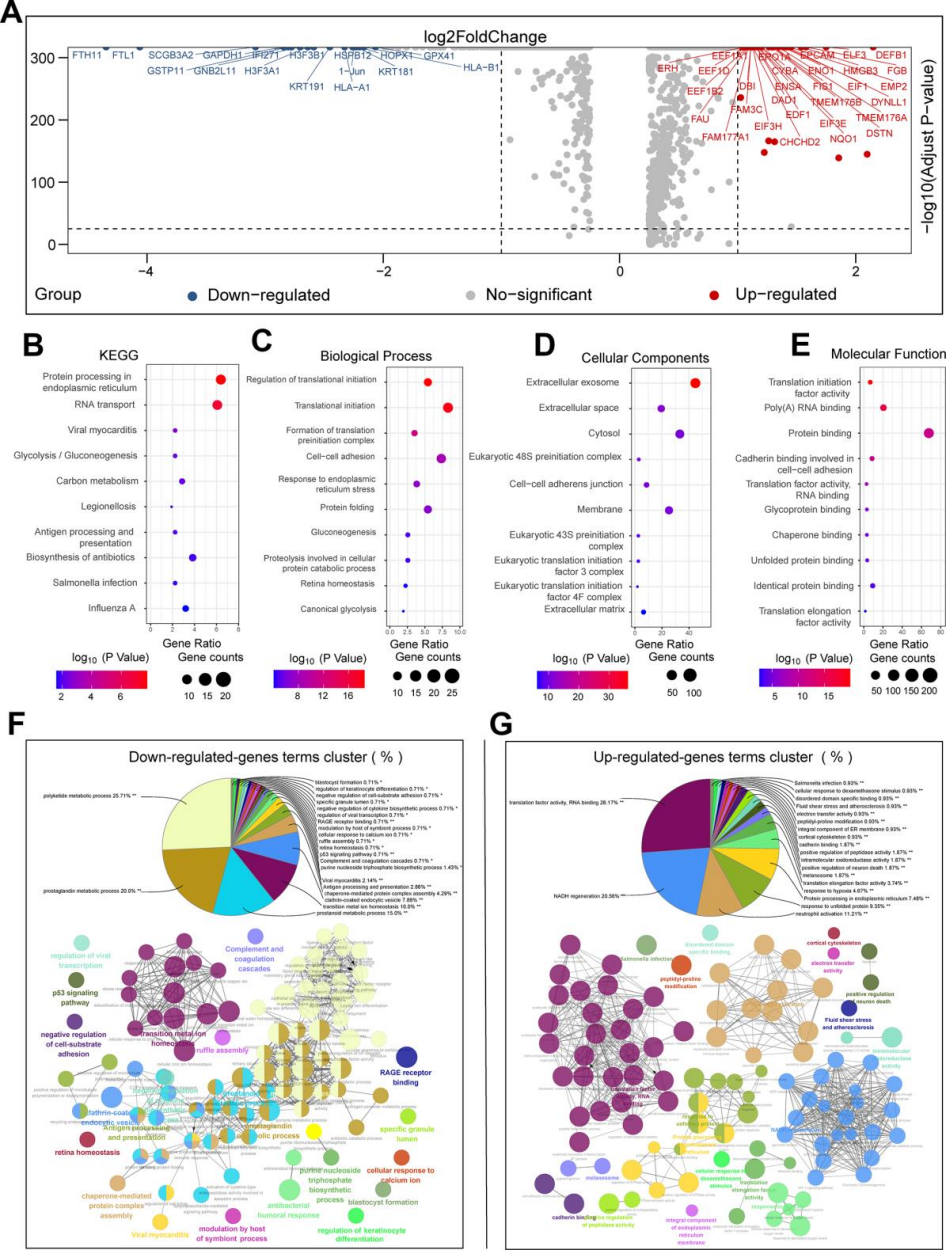
**Functional enrichment analyses of DEGs between primary LUAD and brain metastases without prior treatment.** (**A**) The volcano plot shows the upregulated and downregulated DEGs between primary LUAD tissues and brain metastases tissues without chemotherapeutic treatment. (**B**–**E**) The bubble plots show significantly enriched KEGG pathways, biological processes (BP), cellular components (CC), and molecular functions (MF) based on the analysis of the DEGs between primary LUAD tissues and brain metastases tissues without chemotherapeutic treatment. (**F**) The pie chart and pathway network show the results of functional enrichment analysis of the downregulated genes in primary LUAD tissues and brain metastases tissues without chemotherapeutic treatment. (**G**) The pie chart and the pathway network show the results of the functional enrichment analysis of upregulated genes in primary LUAD tissues and brain metastases tissues without chemotherapeutic treatment.

We performed single-cell transcriptome data analysis on cells from eighteen primary tumor and ten brain metastases samples from LUAD patients without chemotherapeutic treatment to determine tumor heterogeneity at a single cell level. We used the FindMarkers function of Seurat and the Wilcoxon rank sum test to identify differentially expressed genes (DEGs) between cells from primary LUAD and brain metastasis samples. As shown in the volcano plot, we identified 1050 DEGs, including 801 upregulated and 249 downregulated genes in the primary LUAD-derived cells compared to brain metastasis-derived cells using log fold change ≥ 0.25 and adjusted P value <0.05 as the selection criteria (Figure 3A). We then performed Gene Ontology (GO) and Kyoto Encyclopedia of Genes and Genomes pathway (KEGG) enrichment analyses for all the 1050 DEGs (Figure 3B–3E). We separately analyzed the upregulated and downregulated DEGs, and identified the functionally enriched or downregulated biological processes (BP) and signaling pathways in the primary LUAD- and brain metastasis-derived cells (Supplementary Table 2 and Figure 3F, 3G). The pathway enrichment analysis showed that genes involved in translational initiation, endoplasmic reticulum stress, extracellular exosomes, and unfolded protein response were upregulated in the brain metastases samples. The eukaryotic translation initiation factors, namely, EIF1, EIF3E, EIF3H, EIF4G2, EIF5, and EIF6, were all upregulated in the brain metastasis-derived tumor cells. The most upregulated gene in the brain metastases samples was defensin beta 1 (DEFB1). A previous study showed that DEFB1 is a potential diagnostic biomarker for lung cancer [16]. Moreover, enediyne-activated, EGFR-targeted human β-defensin 1 shows therapeutic efficacy against non-small cell lung carcinoma [17]. Our data suggests that DEFB1 is a potential target for treatment of brain metastases in LUAD patients. Survival analysis of the TCGA LUAD patient dataset (n=510) revealed that more than forty upregulated DEGs were associated with poor survival outcomes in LUAD patients (Supplementary Figure 2A). These included annexin A2 (ANXA2), aspartyl-tRNA synthetase (DARS), DEAD-box helicase 41 (DDX41), dehydrogenase/reductase X-linked (DHRSX), egl-9 family hypoxia inducible factor 1 (EGLN1), family with sequence similarity 103 member A1 (FAM103A1), homeobox B7 (HOXB7), p53 apoptosis effector related to PMP22 (PERP), proteasome 26S subunit ATPase 6 (PSMC6), Rac family small GTPase 1 (RAC1), STEAP family member 1 (STEAP1), and voltage dependent anion channel 1 (VDAC1).

We also analyzed the DEGS between chemotherapy-treated and untreated LUAD patients with brain metastases (Supplementary Figure 3A) and identified 1167 DEGs, including 435 upregulated and 732 downregulated genes (Supplementary Table 3). As shown in the bubble charts, DEGs enriched pathways related to extracellular exosome, cell adhesion and metabolic pathways (Supplementary Figure 3B–3E). Moreover, we observed significant enrichment of genes involved in immune response, especially leukocyte activation, which is a positive survival factor that inhibits tumor progression (Supplementary Figure 3F, 3G). The chemotherapy-treated group we analyzed including three brain metastasis patients, all of whom received chemotherapy with different regimens. Two brain metastasis patients were treated with cisplatin and vinorelbine, whereas, the remaining one received erlotinib. It is plausible that the cytotoxicity of chemotherapeutic drugs induces tumor cell apoptosis and releases tumor antigens that provoke immune responses.

Genes related to the extracellular exosome process were upregulated in patients with brain metastasis that did not undergo prior chemotherapeutic treatment compared to those with primary tumors (Figure 3D) and those with brain metastasis that underwent chemotherapy (Supplementary Figure 3D). We then further investigated the potential therapeutic significance of these genes (Supplementary Figure 3H). Clusterin (CLU) is a stress-associated cytoprotective protein up-regulated by various apoptotic triggers in many cancers and confers treatment resistance when overexpressed [18]. In our results, CLU was highly upregulated in the brain metastasis samples from patients that had undergone chemotherapy. We compared CLU expression in tumor cells from different samples and found that CLU expression was highest in tumor cells from the GSM3516671 sample, which received cisplatin +vinorelbine and associated with chemotherapeutic resistance. Therefore, our data suggests that clusterin may be a potential therapeutic target for this patient. Cathepsin Z (CTSZ) was another gene that is upregulated in samples from patients with brain metastasis that have undergone chemotherapy (Supplementary Figure 3H). CTSZ is a target of the antimetastatic drug deguelin, which exerts its anti-metastatic effect by suppressing of CTSZ expression and interrupting the interaction of CTSZ with integrin β3 [19]. Overall, our analysis of differentially expressed genes in single-cell resolution experiments demonstrates significant tumor heterogeneity between primary and metastatic tumors.

### Intertumoural expression profiles across different samples

**Figure 4:**
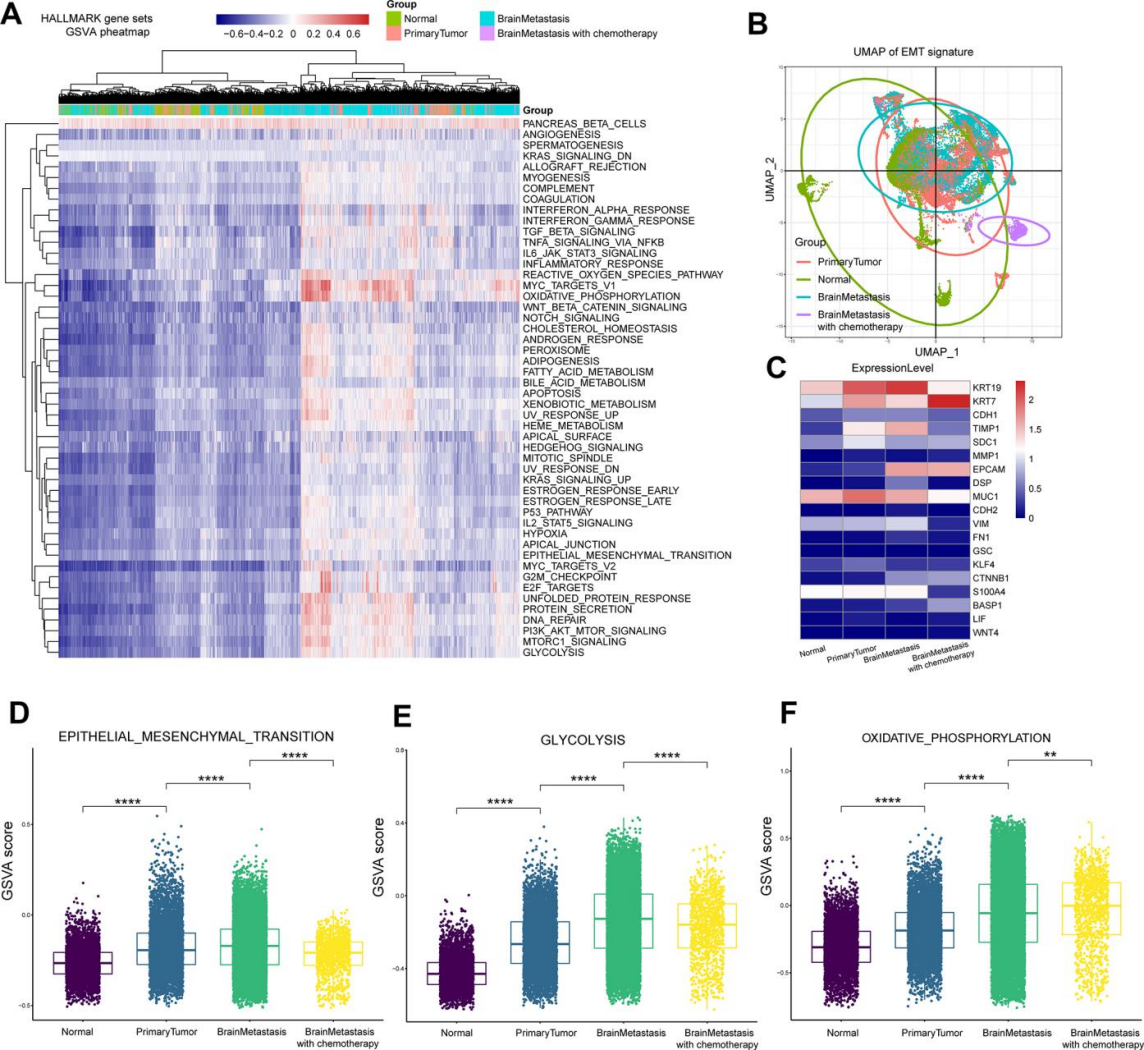
**Gene set variant analysis of LUAD patient samples from primary tumors and brain metastases.** (**A**) Heatmap of 50 cancer hallmark gene sets in primary LUAD and brain metastasis samples. The color index from navy blue to red indicates low to high expression of the gene sets. (**B**) The UMAP graph shows the diversity of EMT gene expression in the primary LUAD and brain metastasis samples at different stages of cancer progression. (**C**) Heatmap shows the mean expression of EMT-associated genes in the primary LUAD and brain metastasis samples at different stages of cancer progression. (**D**) The box plot shows the expression of the EMT pathway genes in the primary LUAD, brain metastasis (with or without chemotherapy) samples. (**E**) The box plot shows the expression of the glycolysis pathway genes in the primary LUAD and brain metastasis (with or without chemotherapy) samples. (**F**) The box plot shows the expression of the oxidative phosphorylation pathway genes in the primary LUAD and brain metastasis (with or without chemotherapy) samples

We used gene set variation analysis (GSVA) and MSigDB to identify specific oncogenic gene set signatures during LUAD progression at a single tumor cell level. We first transformed the observed GSVA scores into binary scores. The heatmap shows the GSVA scores for all cells (Figure 4A). Epithelial-mesenchymal transition (EMT) is a critical process during tumor progression and metastasis, where the tumor cells undergo changes from an epithelial to a mesenchymal phenotype [20]. We analyzed the status of EMT-related genes in different tumor tissues representing early and advanced stages by performing principal component analysis (PCA) of single cells. The uniform manifold approximation and projection (UMAP) [21] graph shows the changes in the expression of EMT-related genes in primary tumor and metastasis samples. The corresponding GSVA scores for different tumor samples are shown in Supplementary Table 4. The EMT gene signature score in the primary LUAD samples was higher than the normal lung tissue samples, whereas, the EMT gene signature score of brain metastasis samples without prior treatment was higher than the primary LUAD samples; moreover, the EMT gene signature score for brain metastasis samples of patients that underwent chemotherapy was lower than samples from brain metastasis patients without prior treatment as well as samples from primary tumors (Figure 4D). To further understand this phenomenon, we analyzed the expression of EMT-associated genes in different tumor groups. Expression of the epithelium-specific cell surface markers epithelial cell adhesion molecule (EPCAM) and keratin 7 (KRT7), and the mesenchymal-specific markers such as matrix metallopeptidase 1 (MMP1), cadherin 2 (CDH2), and vimentin (VIM) were upregulated in samples from patients with brain metastasis without chemotherapy compared to the primary LUAD samples (Figure 4C). These results demonstrate that the EMT process is significantly upregulated in the brain metastasis-related tumor cells compared to the primary LUAD cells. It is reported that that the chemotherapeutic drug vinorelbine inhibits metastasis by downregulating EMT [22]. Therefore, we postulate that chemotherapy with cisplatin and vinorelbine downregulates EMT in brain metastasis cell samples of LUAD patients. Furthermore, glycolysis and oxidative phosphorylation were upregulated in the brain metastasis samples without prior treatment, but glycolysis was downregulated in brain metastasis samples with chemotherapy (Figure 4E–4F).

### Intratumoural expression heterogeneity

**Figure 5:**
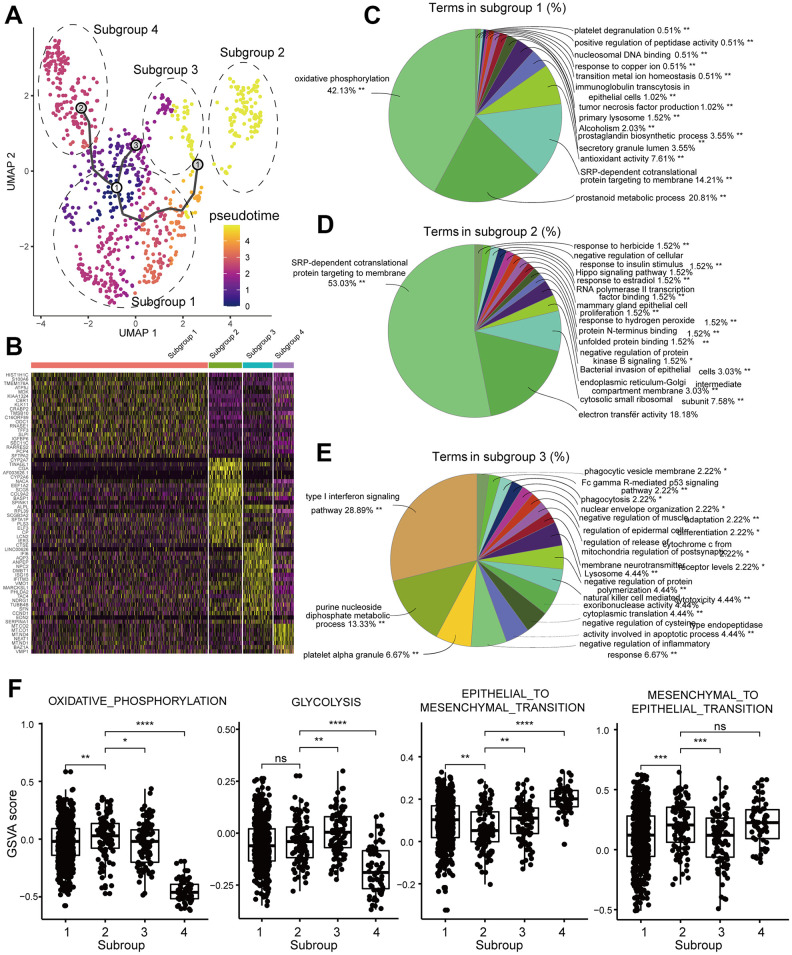
**Functional enrichment analyses of the DEGs in the tumor cell subgroups of the brain metastasis sample, GSM3516671.** (**A**) UMAP plot shows the different trajectories of the four tumor cell subgroups in GSM3516671. (**B**) Heatmap shows the differentially expressed genes (DEGs) in the four tumor cell subgroups of the brain metastasis sample, GSM3516671. (**C**–**E**) Pie graphs show the enrichment analysis for the three tumor cell subgroups of the brain metastasis sample, GSM3516671 (DEGs in the subgroup 4 were not sufficient for enrichment analysis). (**F**) Gene set variation analysis (GSVA) of the 4 tumor cell subgroups in the brain metastasis sample, GSM3516671.

**Figure 6:**
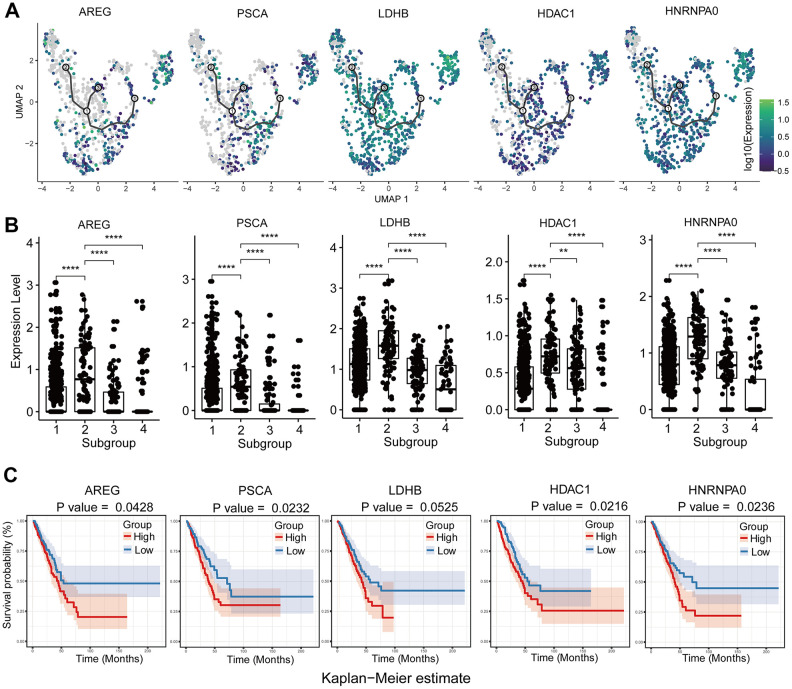
**Analysis of upregulated genes in GSM3516671-subgroup 1 cells associated with chemotherapy resistance.** (**A**) Increased expression of chemotherapy resistance-associated genes with pseudotime extension of single cell trajectories. (**B**) Comparative analysis of the expression of chemotherapy resistance-associated genes in subgroups 1-4 of the GSM3516671 sample. (**C**) Survival analysis based on the high- or low-expression of these chemotherapy resistance-related genes in the TCGA LUAD dataset (n=510).

Next, we assessed heterogeneity among tumor cells within the same tumor sample by clustering cells into different subgroups based on their differential gene expression using Seurat. Then, we used Monocle3 to construct single cell trajectories and the FindAllMarkers function to analyze distinct gene expression patterns for each subgroup of tumor cells in a single tumor (Figure 5A). We analyzed DEGS for each of the 4 subgroups (subgroup 1-4) in the brain metastasis sample GSM3516671 (Figure 5B). The enriched GO terms and KEGG pathways in the four different subpopulations are shown in Figure 5C–5E. We then performed GSVA for each subgroup (Figure 5F). The GSM3516671 subgroup 1 with 548 cells out of a total of 806 cells in the sample showed upregulation of oxidative phosphorylation, prostanoid metabolic process and prostagladin biosynthetic process. Prostanoid biosynthesis and metabolic processes are related to chemotherapy response and induction of tumor cell repopulation [23]. The GSM3516671 subgroup 2 was enriched in genes related to ribosome and mitochondrial functions. However, we also found that several upregulated genes were related to chemotherapy resistance in subgroup 1 and were associated with poor prognosis in LUAD patients of the TCGA dataset based on the survival analysis (Figure 6A–6C). Moreover, the functional annotation of GSM3516671-subgroup 3 mostly showed enrichment of genes related to immune responses, such as type I interferon signaling pathway, negative regulation of inflammatory response, and natural killer cell mediated cytotoxicity (Figure 5E). This indicates that tumor cells from GSM3516671-subgroup 3 were recognized and attacked by the immune system. However, the number of DEGs analyzed in GSM3516671-subgroup 4 was too small to perform enrichment analysis.

**Figure 7:**
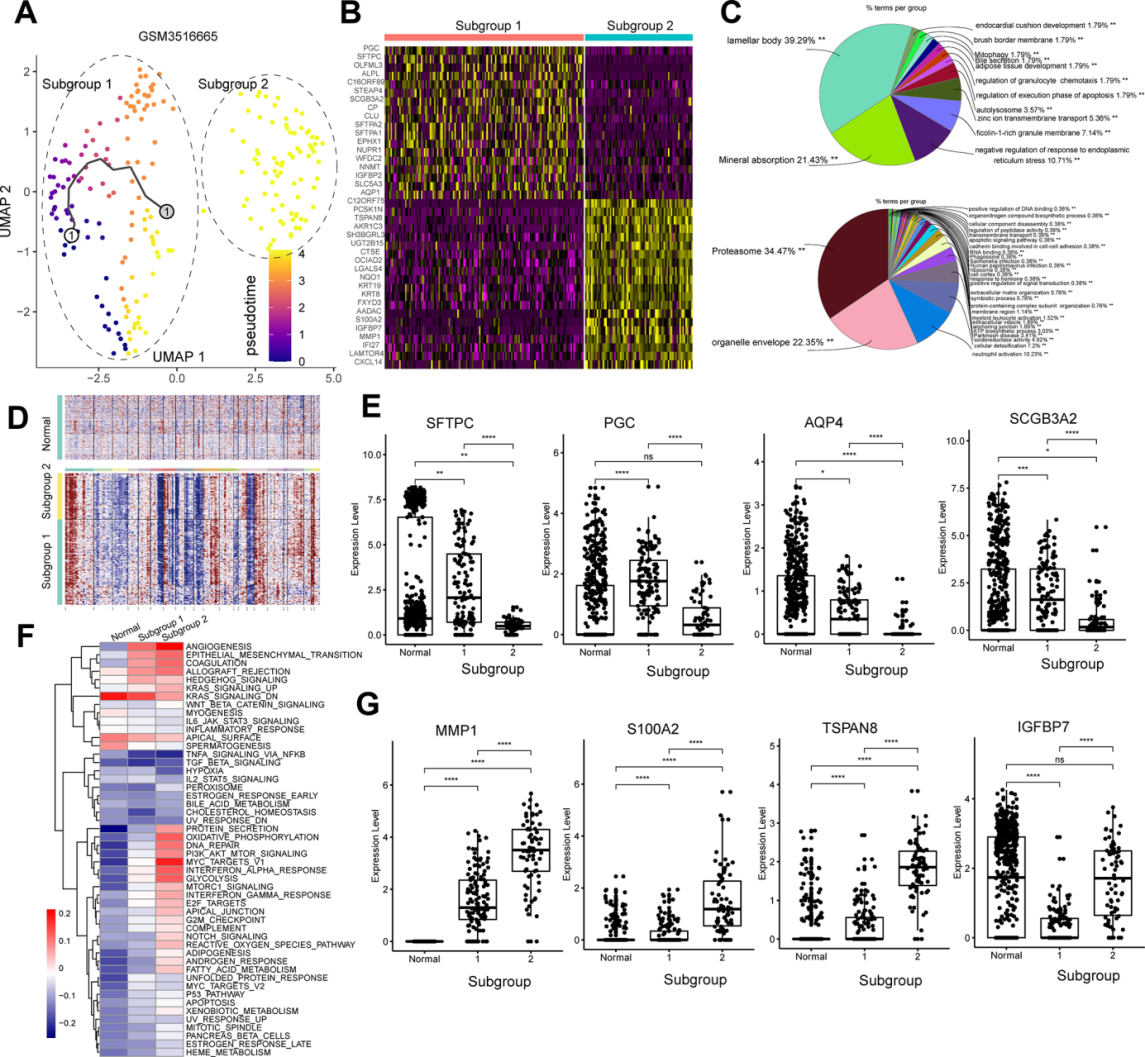
**Analysis of the tumor cell subgroups in the stage IV primary LUAD sample, GSM3516665.** (**A**) UMAP plot shows the different trajectories of two tumor cell subgroups from the GSM3516665 sample. (**B**) Heatmap shows the DEGs between the two tumor cell subgroups from the GSM3516665 sample. (**C**) Pie graphs show the results of the functional enrichment analysis of the DEGs between the two tumor cell subgroups from the GSM3516665 sample. (**D**) InferCNV plot shows significant copy number variations in the chromosomes of the two tumor cell subgroups from the GSM3516665 sample in comparison with the normal lung epithelial cells. (**E**) Heatmap shows the gene set variation analysis of the two subgroups from the GSM3516665 sample and the normal lung epithelial cells. The color code in the heat maps ranges from navy blue to red and shows progression from low to high expression of the gene sets. (**F**) Gene expression analysis shows that genes associated with the normal type II alveolar Type cells such as *SFTPC, PGC, AQP4,* and *SCGB3A2* are upregulated in the subgroup 1 cells from the GSM3516665 sample. (**G**) Gene expression analysis shows that genes associated with tumor progression such as *MMP1, S100A2, TSPAN8*, and *IGFBP7* are upregulated in the subgroup 2 from the GSM3516665 sample.

We also investigated GSM3516665, the stage IV primary tumor sample. We re-clustered the GSM3516665 primary tumor sample to 2 subgroups. Then, we performed functional enrichment analysis of the DEGs in the subgroups of cells from GSM3516665 (Figure 7B). We obtained only two subpopulations in the GSM3516665 sample (Figure 7A). GSM3516665-subgroup 1 was enriched in genes related to lamellar bodies and mineral absorption, whereas, GSM3516665-subgroup 2 was enriched in genes related to proteasomes and organelle envelope (Figure 7C). This demonstrates differences in the activation of the biological processes in the two different tumor cell subgroups from the same tumor tissue. Under normal circumstances, lamellar bodies are characteristic of the type II pulmonary alveolar cells [24]. We found upregulation of several genes associated with type II pulmonary alveolar cells, including surfactant protein C (SFTPC), progastricsin (PGC), aquaporin 4 (AQP4), secretoglobin family 3A member 2 or SCGB3A2 [25, 26] in GSM3516665-subgroup 1 (Figure 7E). The results of the inferCNV analysis excluded the possibility of subgroup 1 as normal alveolar Type II cells (Figure 7D). GSM3516665-subgroup 2 was enriched in genes related to metabolic pathways and tumor progression, such as EMT, angiogenesis, oxidative phosphorylation (OXPHOS), and glycolysis (Figure 7F). Genes related to tumor progression, including matrix metallopeptidase 1 or MMP1 [27], S100 calcium binding protein A2 or S100A2 [28], tetraspanin 8 or TSPAN8 [29], and insulin like growth factor binding protein 7 or IGFBP7 [30] were upregulated in GSM3516665-subgroup 2 (Figure 7G).

We then analyzed the tumor cells from the bone metastasis sample GSM3516664, and the adrenal metastasis sample GSM3516677. However, we identified only 14 tumor cells from GSM3516677 and did not perform the downstream functional enrichment analysis. We re-clustered the GSM3516664 bone metastasis sample and compared the gene expression profiles with those of the primary tumors and brain metastases samples in GSE123902 and GSE131907 using GSVA. We identified 659 tumor cells from the GSM3516664 sample and divided them into 3 subgroups based on their gene expression trajectories (Supplementary Figure 4A). GSVA analysis showed that cancer hallmark gene sets related to the inflammatory response, IL6-JAK-STAT3 signaling, and allograft rejection were upregulated (Supplementary Figure 4B). We also analyzed the bone metastasis sample, GSE123902, which was isolated from the LUAD patient that received chemotherapy. The results showed that EMT process was upregulated in GSE123902 unlike the brain metastases samples from LUAD patients treated with chemotherapy, thereby suggesting activation of EMT-induced chemo-resistance mechanisms (Supplementary Figure 4C). Moreover, in the bone metastasis sample (GSM3516664), glycolysis was upregulated (Supplementary Figure 4D) and oxidative phosphorylation was downregulated (Supplementary Figure 4E). This suggests that glycolysis is the predominant metabolic pathway in the bone metastasis cells from LUAD patients.

## DISCUSSION

Several investigations are underway to understand the factors and mechanisms regulating tumor heterogeneity in order to design personalized and optimal targeted therapies [31]. Inter- and intra-tumoral heterogeneity is closely related to tumor progression and metastasis and influences the intrinsic biological characteristics of tumors that determine the diagnosis, response to targeted therapies, and eventually survival outcomes. The resolution achieved by single-cell RNA sequencing technology involves assessing the gene expression profiles of individual tumor cells in a tumor sample, which allows identifying different cell clusters, novel tumor drivers, responses to therapies, and new therapeutic targets. In this study, we performed single-cell transcriptome analysis to characterize the tumor heterogeneity in the primary lung adenocarcinoma and brain metastases samples. We found distinct differences in the biological processes that are active in the primary tumors compared to the metastatic tissues at advanced stages. We also identified tumor cell subgroups within the same tumor sample with differential cellular responses to chemotherapy. We also identified distinct cell subgroups in metastatic tissues with gene set signature associated with drug resistance.

Previous studies show that exosomal proteins and noncoding RNAs including long noncoding RNAs (lncRNAs) and microRNAs (miRNAs) promote survival of cancer cells, mediates intracellular communication during cancer metastasis, and modulates drug resistance and immune responses [32, 33]. Our study demonstrates that exosome-related genes are upregulated in the brain metastases-related cells compared to the primary LUAD-related cells. Moreover, upregulation of exosome-related genes correlates with poor survival times of LUAD patients in the TCGA dataset. In our study, brain metastasis samples show association between the upregulation of several exosomal markers and various cancer hallmarks (Supplementary Figure 3H). For example, clusterin (CLU) is associated with chemoresistance and tumor proliferation in pancreatic cancer [34]; insulin like growth factor binding protein 2 or IGFBP2 [35] is related to signaling pathways associated with tumor cell migration; heat shock protein 90 beta family member 1 or HSP90B1 [36] correlates with poor prognosis and lymph node metastases in melanoma. These upregulated genes in the brain metastasis samples indicate that exosomes carry different oncogenic proteins or RNAs that modulate proliferation, progression, stemness, chemoresistance, and brain metastasis. Members of the carcinoembryonic antigen related cell adhesion molecule (CEACAM) family including CEACAM6 modulate immune response, tumor progression, metastasis and angiogenesis [37]. Exosomal MIF levels are significantly higher in the stage I pancreatic ductal adenocarcinoma patients who eventually develop liver metastasis [38].

One of the most striking characteristics of tumor cells is their ability to alter metabolic pathways as an adaptation to the changing environmental conditions in order to utilize a wide range of nutrients [39]. Since reprogramming of metabolic pathways is an essential feature of tumor cell growth and progression, key metabolic genes and activities are targets for effective cancer therapy [40]. Tumor cells prefer to utilize glycolysis for their energy needs even in oxygen-rich conditions [41]. Cisplatin resistance involves metabolic reprogramming through modulation of the ROS and PGC-1α signaling pathways in NSCLC cell lines, but, treatment with OXPHOS inhibitors such as metformin or rotenone improves cisplatin sensitivity [42]. In our study, gene set variation analysis showed that chemotherapeutic treatment resulted in a switch to oxidative phosphorylation in a subset of tumor cells derived from brain metastases samples GSM3516671 (Figure 4E–4F).

The gene encoding surfactant protein C (SFTPC) is deleted in 71% of the NSCLC tumor tissues [43]. Moreover, SFTPC knockdown promotes lung adenocarcinoma progression [44]. SFTPC expression was significantly reduced in both subgroups of cells from the stage IV primary tumor sample, GSM3516665. Analysis of the expression of other marker genes suggests that GSM3516665 might have originated from the Type II alveolar cells. Furthermore, SFTPC expression is lower in the GSM3516665-subgroup 2 compared to GSM3516665-subgroup 1. This suggests that the GSM3516665-subgroup 2 tumor cells are more progressed than the GSM3516665-subgroup 1 tumor cells. Moreover, tumor cells from this stage IV sample show upregulation of genes related to EMT, angiogenesis, and metabolic pathways, including those that have been previously associated with tumor progression such as *MMP1*, *S100A2*, *TSPAN8*, and *IGFBP2*.

We also analyzed chemotherapy-induced transcriptome changes in the subgroups of brain metastasis-derived tumor cells from patients that underwent chemotherapy. Genes related to oxidative phosphorylation and those related with chemoresistance and poor prognosis such as HNRNPA0, HDAC1, LDHB, AREG, and PSCA were upregulated in subgroups of brain metastasis-derived tumor cells in chemotherapy treated patients (Figure 6A–6C). High expression of HDAC1 mRNA is associated with multidrug resistance in the neuroblastoma cell lines [45]. Moreover, activation of histone deacetylases (HDACs) promotes proliferation and progression of paclitaxel-resistant NSCLC cells [46]. Moreover, inhibition of HDAC1 improves gefitinib sensitivity in the gefitinib-resistant NSCLC cells [47]. Our study shows that high HDAC1 expression is related to poor prognosis in the LUAD patients from the TCGA dataset (Figure 6C). The brain metastasis samples from LUAD patients that received cisplatin+vinorelbine demonstrate upregulation of HDAC1 and downregulation of the HDAC1 target gene, CDKN1A or P21. This suggests that treatment with cisplatin+vinorelbine or paclitaxel induces upregulation of HDAC1 and is associated with the resistance of metastatic cells to cisplatin+vinorelbine. The subgroup 1 cells from the brain metastasis sample shows higher expression of genes related to OXPHOS than those related to glycolysis. However lactate dehydrogenase B (LDHB), a key glycolytic enzyme, is also upregulated in the subgroup1 cells. LDHB catalyzes the interconversion between pyruvate and lactate and determines the chemotherapy response and prognosis in oral squamous cell carcinoma and breast cancer patients [48, 49]. Therefore, LDHB upregulation may indicate chemoresistance, and upregulating of glycolysis and OXPHOS may be associated with metabolic alterations in tumor cells in response to chemotherapy. Osimertinib overcomes alectinib resistance caused by increased amphiregulin (AREG) expression in the leptomeningeal carcinomatosis (LMC) model of ALK-rearranged lung cancer; AREG levels are also significantly higher in the cerebrospinal fluid of patients with alectinib-resistant ALK-rearranged NSCLC with LMC [50]. The overexpression of EGFR ligands such as AREG is associated with crizotinib resistance [51]. We did not detect any EGFR mutations in the brain metastasis samples, but, we detected AREG upregulation in GSM3516671 subgroup 2 (Figure 6B). This suggests that amphiregulin induces resistance to cisplatin or vinorelbine. The upregulation of HNRNPA0 and PSCA is also associated with cisplatin resistance in lung cancer [52]. The upregulation of these genes in subgroup 1 suggests activation of multiple chemoresistance-related mechanisms.

We performed gene set variation analysis of all subgroups of LUAD cells (Figure 5F). We observed consistent upregulation of OXPHOS and glycolysis, especially in the metastatic tumor samples without chemotherapy including brain metastases and bone metastasis samples (Figure 4D, 4E). However, epithelial to mesenchymal transition (EMT) and mesenchymal to epithelial transition (MET) varied among the different subgroups, probably influenced by factors such as chemotherapeutic treatment response and drug resistance. TGF-β-ID1 signaling inhibits Twist1 and promotes metastatic colonization via MET in breast cancer, whereas ID1 induces MET in metastatic cells during lung colonization [53]. We observed ID-1 upregulation in subgroup 1, but changes in TGFB1 and TWIST1 levels were not significant because of poor raw data quality. This phenomenon wherein the carcinoma cells switch partially from an epithelial to a mesenchymal phenotype is called “partial-EMT”. Partial EMT probably occurs because the invasive tumor cells need to establish colonies in the metastasized tissues and develop macrometastatic outgrowths through re-epithelialization process [54]. Combined with multiple marker genes of chemotherapy resistance, we speculate that subgroup 1 cells were chemoresistant and underwent MET for metastatic colonization. However, tumor cells from the bone metastasis samples that received chemotherapy were significantly enriched for genes related to EMT, glycolysis and hypoxia compared the brain metastases samples without chemotherapy (Supplementary Figure 4C). Tumor microenvironment plays an important role in changing the biological phenotypes of tumor cells. Activation of the PI3K/Akt/HIF-1α pathway contributes to hypoxia-induced EMT and chemoresistance in hepatocellular carcinoma [55]. Moreover, cancer-associated fibroblasts contribute to cancer cell survival and drug resistance [56]. Our study suggests an association between metabolism, cancer-associated fibroblasts, EMT and chemoresistance, but further experiments were needed to confirm these findings.

In summary, we used single-cell RNA sequencing data analysis to elucidate the landscape of lung adenocarcinoma heterogeneity in the primary tumors and brain metastases. Our results demonstrate single cell transcriptional profiles that correlate with the status of tumor progression and the chemotherapeutic response of each patient. We demonstrate metabolic reprogramming including aberrant regulation of OXPHOS and glycolysis, upregulation and activation of the exosomes, upregulation of chemoresistant genes, and partial-EMT by comparing the gene expression profiles of cells derived from primary and metastatic tumor tissues. Our study cannot eliminate the possibility of bias because we analyzed cells from primary tumors and brain metastases from different patients. Overall, our study demonstrates that single cell transcriptome analysis is a useful tool for accurate diagnosis and personalized targeted treatment of LUAD patients.

## MATERIALS AND METHODS

### Data processing

### RNA-seq datasets of LUAD patient samples

We downloaded the single-cell RNA transcription data from the GSE123902 [14] and GSE131907 [15] LUAD patient datasets in the Gene Expression Omnibus (GEO) database. This included single-cell RNA-seq data from 23 human primary LUAD tissue samples, 15 non-tumor lung samples, and 13 brain, 1 bone, and 1 adrenal samples of lung adenocarcinoma metastasis. The 10X Genomics Chromium Platform was used to generate a targeted 5000 single-cell Gel Bead-In-Emulsions (GEMs) per sample. The libraries from single cells were then sequenced on an Illumina HiSeq2500 system according to the 10X Genomics protocol. The raw expression matrix was downloaded and imported into the Seurat (v3.1.4) R package [57] for downstream analysis. Quality control and filtration of datasets was performed to remove the data from low quality cells and isolate cells with high-quality data. The raw counts were normalized using the LogNormalize method in the NormalizeData function of the Seurat package, and highly variable genes were identified using the FindVariableFeatures function in all datasets.

We also downloaded the raw counts of the RNA-seq expression data from 510 lung adenocarcinoma patient tissue samples and the corresponding clinical information from the TCGA database [58].

### Dataset integration and joint analysis

We performed routine data integration process on the two datasets GSE123902 and GSE131907. The primary lung adenocarcinoma sample mixed with large cell neuroendocrine carcinoma in GSE123902 was excluded from the analysis. The remaining 7 primary tumor samples, 3 brain metastasis samples and 4 non-tumor lung samples from GSE123902 and 15 primary tumor samples, 10 brain metastasis samples and 11 non-tumor lung samples from GSE131907 were integrated for joint analysis. The integrated Seurat object was used to obtain batch-corrected gene expression matrix for all cells. Then, the joint data analysis was performed and visualized. Then, a linear transformation function called ScaleData was used to ensure that expression of all genes were given equal weight in the downstream analyses and highly expressed genes were not dominant. Next, principal component analysis (PCA) was performed for 50 principal components. The FindNeighbors and FindClusters functions were used for cell clustering with a graph-based method (resolution=5.0). We used the nonlinear dimensionality reduction technique called t-distributed stochastic neighbor embedding (tSNE) to visualize the clusters with the top 50 principal components.

### Cell type identification

The Single R package [59] was used to annotate every single cell in the cell clusters described above. Spearman correlation analysis was used to compare the cell expression profiles with that of each reference cell sample. The per-label score was defined as a fixed quantile (0.8) of the distribution of correlations. Spearman correlation analysis was performed between unknown cells from our samples and reference cells from reference data, and reference cells with the highest per-label score was used for the annotation of each unknown cells. Reference data for cell annotation was obtained from the Human Primary Cell Atlas [60], Blueprint [61] and the encyclopedia of DNA elements or ENCODE [62]. The log-count matrix was generated from Seurat and imported into Single R for cell annotation. The cells from the non-tumor, primary tumor, and brain metastasis samples annotated as epithelial cells (including tumor cells) were integrated and re-clustered using Seurat. Then, the data was imported to the inferCNV R package for further analysis.

### Tumor cell identification and isolation

Single R package identified tumor cells as epithelial cells. Therefore, we integrated all the epithelial cells from the LUAD and normal lung tissue samples for re-clustering, and the principal component analysis was used to identify cell clusters with a 1.0 resolution using the original Louvain algorithm and visualized using the t-SNE algorithm. The clusters with majority of the cells from normal lung tissues were categorized as normal lung epithelial cells and the remaining clusters were categorized as tumor cells. Then, we used the inferCNV R package [63] to assess accuracy of clustering. InferCNV compared the genome-wide expression of the tumor cell genes with a set of reference cells in order to identify large-scale chromosomal copy number variations (CNVs). The resulting CNVs were used to determine the accuracy of cell clustering classification into tumor cell clusters and normal epithelial cells. The mean expression threshold was set as 0.1for the 10X Genomics single-cell datasets. We also analyzed several epithelial tumor marker genes using Seurat and SingleCellSignatureExplorer including EPCAM, CDH1, KRT7, SLPI, MUC1, WFDC2. The raw expression matrix generated from Seurat was used as input data for the inferCNV analysis. Pulmonary epithelial cells isolated from the normal lung tissue samples were used as reference cells.

### Analysis of differentially expressed genes (DEGs) and functional enrichment

FindMarkers function from the Seurat package was used to analyze the DEGs between primary and metastatic tumors using log fold change (logFC) ≥0.25 according to the Wilcoxon rank sum test and an adjusted p value < 0.05 according to the Bonferroni correction test as threshold parameters. Functional enrichment analysis of the DEGs was performed to identify the significantly enriched Kyoto Encyclopedia of Genes and Genomes (KEGG) pathways [64] and Gene Ontology (GO) terms [65] using the ClueGO and CluePedia plug-ins in Cytoscape [20].

### Gene set variant analysis (GSVA) and single cell trajectory construction

The Gene Set Variation Analysis (GSVA) was performed with the R package [66] for each cell with selected gene sets from the Molecular Signatures Database or MSigDB [67]. The GSVA results were visualized using the SingleCellSignatureExplorer R package [25]. Monocle3 was used to construct single cell trajectories [68–70] and the results were visualized using UMAP [21].

## Supplementary Material

Supplementary Figures

Supplementary Table 1

Supplementary Table 2

Supplementary Table 3

Supplementary Table 4
