# Supplementary material for: Single-cell transcriptome analysis demonstrates inter-patient and intra-tumor heterogeneity in primary and metastatic lung adenocarcinoma
Source: Aging (Albany NY). 2020 Nov 10;12(21):21559–81. doi: 10.18632/aging.103945 (PMC7695431; doi:10.18632/aging.103945)
Supplement: Supplementary Figures [file aging-12-103945-s001..pdf]

SUPPLEMENTARY FIGURES

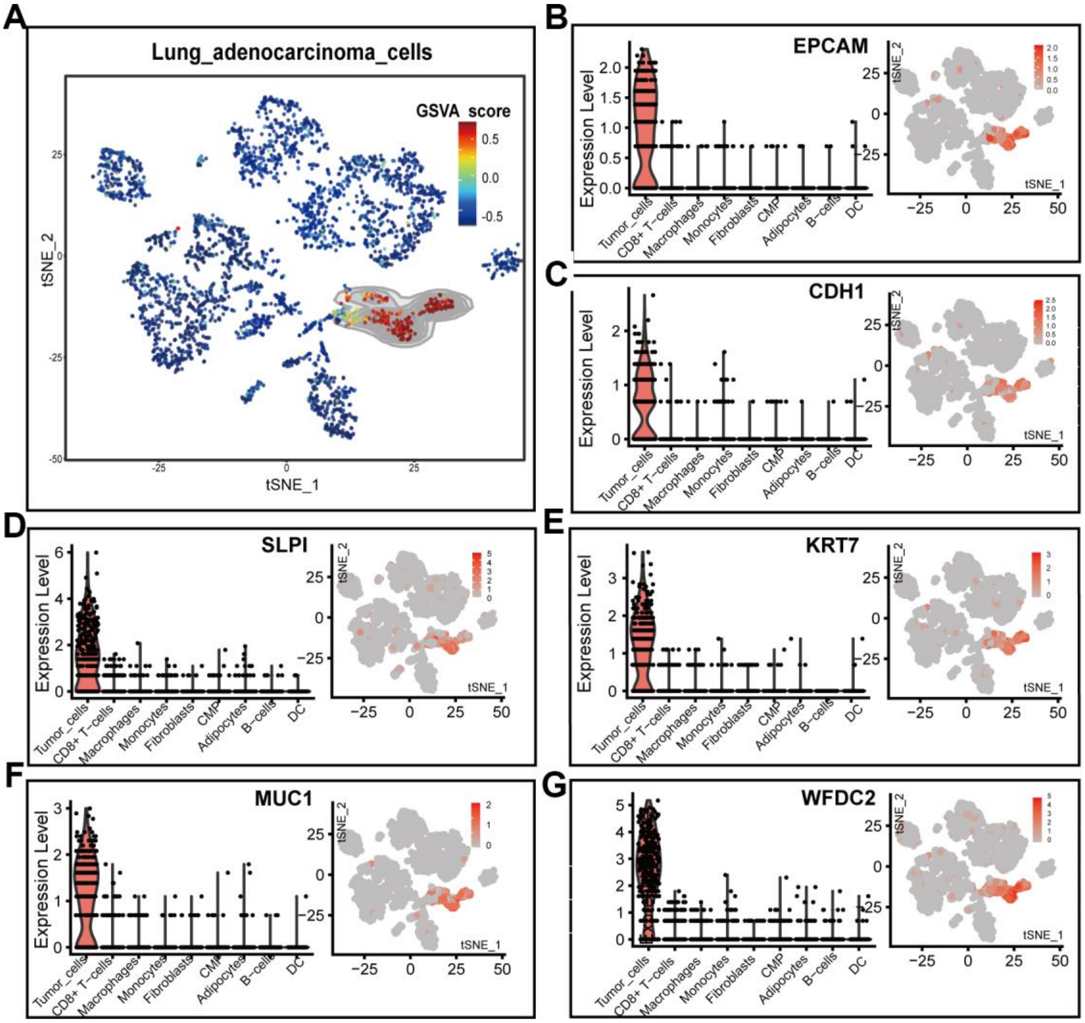

Supplementary Figure 1. Gene expression analysis of the epithelial tumor marker genes in the epithelial cells derived from normal lung epithelium, primary LUAD and metastatic LUAD tissues. (**A**) SingleCellSignatureExplorer plot shows the GSVA results of the expression of epithelial tumor marker genes in cells derived from the normal lung epithelium, primary LUAD and brain metastatic LUAD tissues. (**B**–**G**) Violin plots and t-SNE feature plots show the expression of epithelial tumor marker genes in the tumor cells derived from primary LUAD and brain metastatic LUAD tissues.

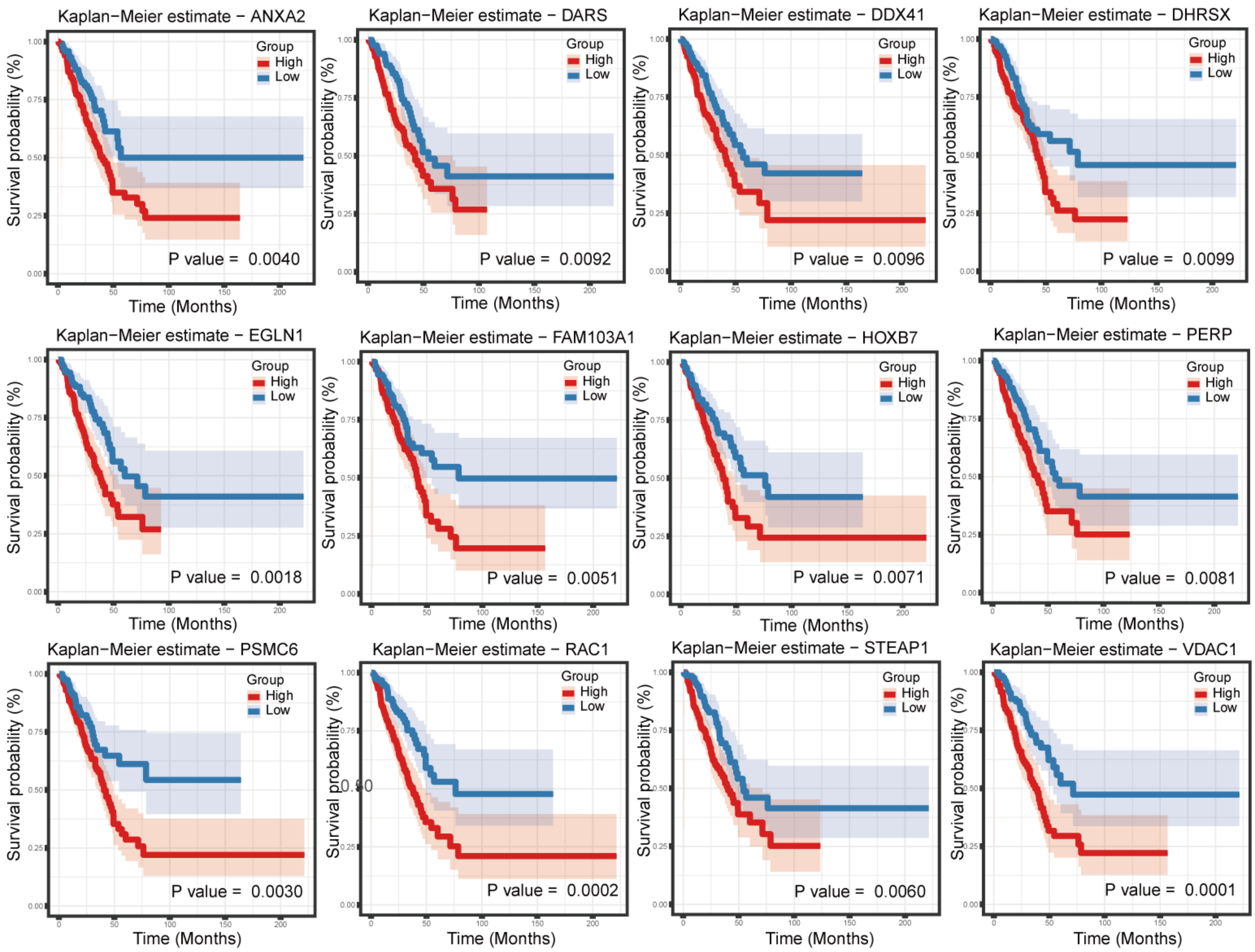

Supplementary Figure 2. Survival analysis of the lung adenocarcinoma patients from the TCGA dataset based on the expression of the upregulated DEGs in the primary LUAD and metastatic LUAD tissues. The results show that 12 upregulated DEGs are associated with poor survival outcomes in the TCGA LUAD dataset (n=510).

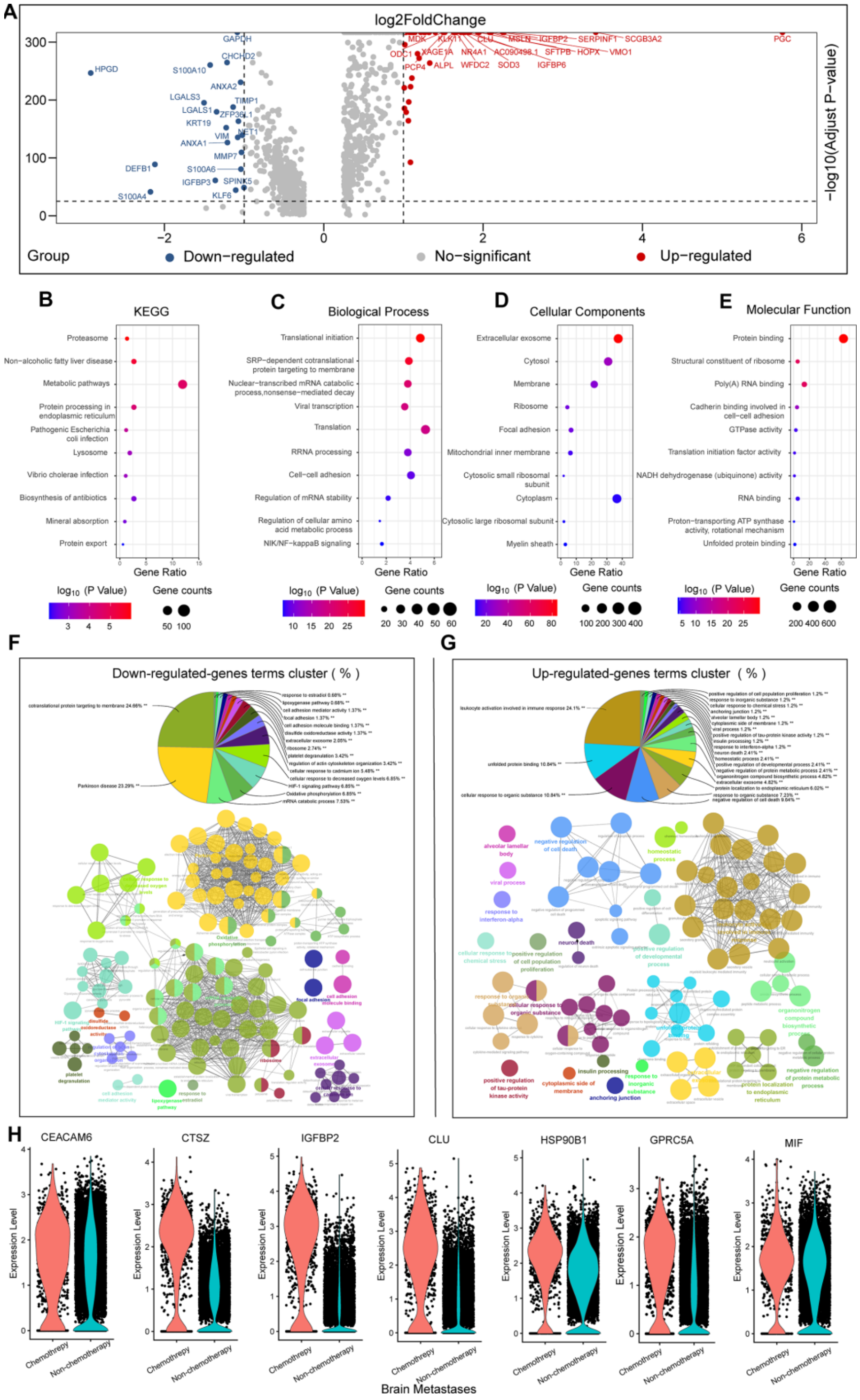

Supplementary Figure 3. Differentially expressed genes (DEGs) between brain metastatic tissues from untreated and chemotherapy-treated LUAD patients and their functional enrichment analysis. (**A**) The volcano plot shows the DEGs between brain metastatic tissue samples from untreated and chemotherapy-treated LUAD patients. (**B**–**E**) Bubble plot shows the significantly enriched KEGG pathways, biological processes (BP), cellular components (CC), and molecular functions (MF) based on the functional enrichment analysis of the DEGs between brain metastatic tissue samples from untreated and chemotherapy-treated LUAD patients. (**F**) Pie graph and pathway network shows the functional enrichment analysis results of the downregulated DEGs between brain metastatic tissue samples from untreated and chemotherapy-treated LUAD patients. (**G**) Pie graph and pathway network shows the functional enrichment analysis results of the upregulated DEGs between brain metastatic tissue samples from untreated and chemotherapy-treated LUAD patients. (**H**) The upregulated DEGs in the brain metastases samples from patients treated with chemotherapy are associated with immune response.

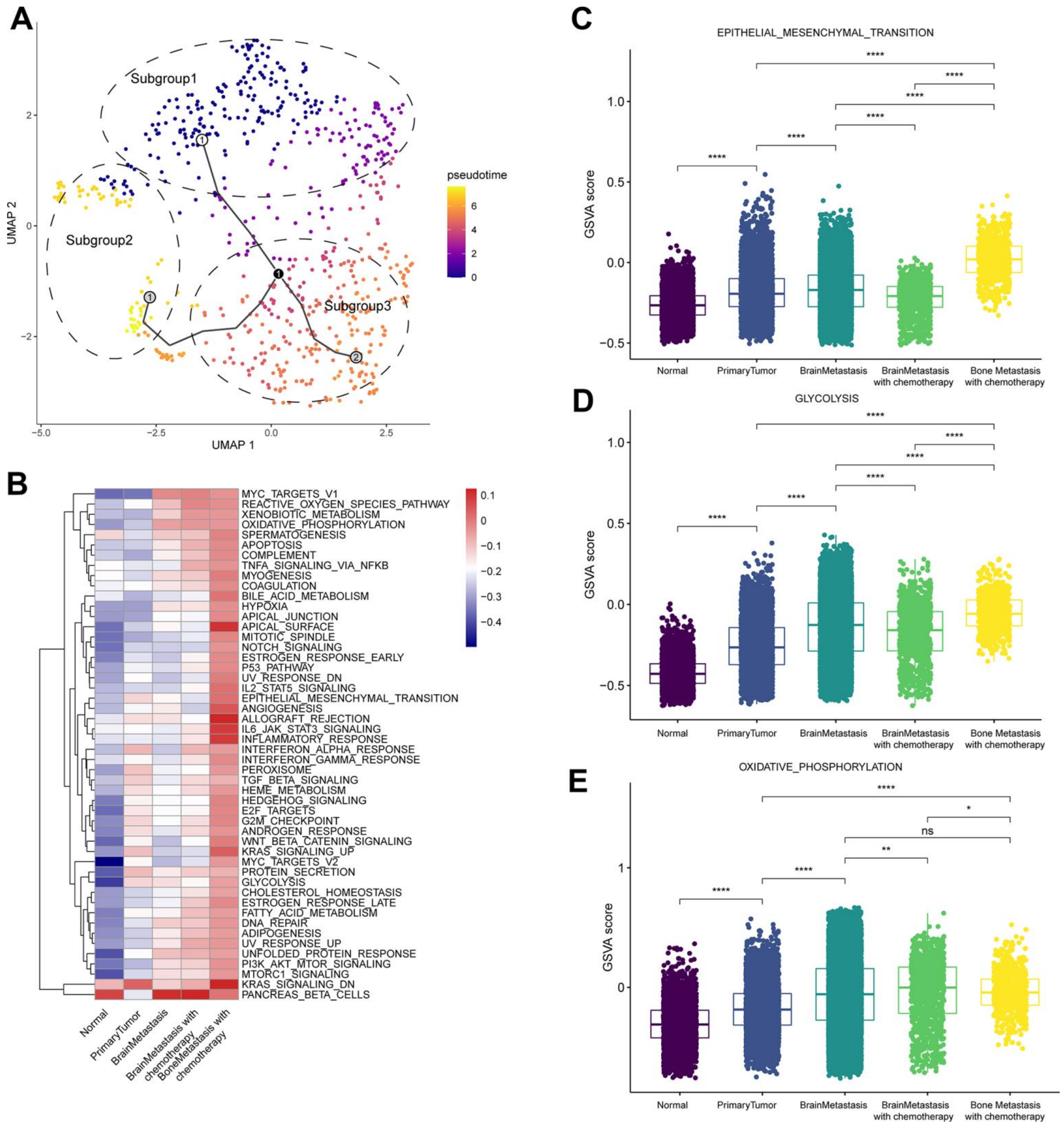

Supplementary Figure 4. Gene expression analysis of bone metastasis sample GSM3516664 from GSE123902 and comparison with the other samples from LUAD patients (GSE123902 and GSE131907). (**A**) UMAP plot shows the trajectories of different subgroups in the bone metastasis sample GSM3516664. (**B**) Heatmap shows the gene set variation analysis (GSVA) for the primary tumor, brain metastases and bone metastasis LUAD patient samples. (**C**) Box graph shows the status of the EMT process in the primary tumor, brain metastases and bone metastasis LUAD patient samples. (**D**) Box graph shows the status of the glycolysis process in primary tumor, brain metastases and bone metastasis LUAD patient samples. (**E**) Box graph show the status of oxidative phosphorylation in primary tumor, brain metastases and bone metastasis LUAD patient samples.
