# Supplementary material for: Single-cell transcriptome analysis demonstrates inter-patient and intra-tumor heterogeneity in primary and metastatic lung adenocarcinoma
Source: Aging (Albany NY). 2020 Nov 10;12(21):21559–81. doi: 10.18632/aging.103945 (PMC7695431; doi:10.18632/aging.103945)
Supplement: Supplementary Table 1 [file aging-12-103945-s002..pdf]

Supplementary Table 1. Comparison methods between primary tumours and brain metastases.

Lung adenocarcinoma		Normal	Primary tumour	Brain metastasis	Brain metastasis with chemotherapy
**chemotherapy**		**No**	**No**	**No**	**Yes**
Intertumoural heterogeneity	DEGs		Enrichment analysis (BP, CC, MF, KEGG)<br>Survival analysis		
	GSVA (Table S3)	Control group	Angiogenesis etc.	Epithelial mesenchymal transition etc.	Oxidative phosphorylation etc.
Intratumoural heterogeneity	DEGs	None	Enrichment analysis in subgroups		
	GSVA	None	Gene sets analysis in subgroups		
	Monocle	None	Single cell trajectories between subgroups		
